# Plasminogen activation by staphylokinase enhances local spreading of *S. aureus* in skin infections

**DOI:** 10.1186/s12866-014-0310-7

**Published:** 2014-12-17

**Authors:** Marijke Peetermans, Thomas Vanassche, Laurens Liesenborghs, Jorien Claes, Greetje Vande Velde, Jakub Kwiecinksi, Tao Jin, Bart De Geest, Marc F Hoylaerts, Roger H Lijnen, Peter Verhamme

**Affiliations:** Center for Molecular and Vascular Biology, KU Leuven, Herestraat 49, Box 911, Leuven, Belgium; Biomedical MRI/Molecular Small Animal Imaging Center, KU Leuven, Herestraat 49, Box 505, Leuven, Belgium; Department of Rheumatology and Inflammation Research, University of Gothenburg, Guldhedsgatan 10, Box 480, Gothenburg, Sweden

**Keywords:** Fibrinolysis, Matrix metalloproteinases, Plasminogen, *Staphylococcus aureus*, Staphylokinase, Skin infection

## Abstract

**Background:**

*Staphylococcus aureus (S. aureus)* is a frequent cause of skin and soft tissue infections. A unique feature of *S. aureus* is the combined presence of coagulases that trigger fibrin formation and of the plasminogen activator staphylokinase (SAK). Whereas the importance of fibrin generation for *S. aureus* virulence has been established, the role of SAK remains unclear.

We studied the role of plasminogen activation by SAK in a skin infection model in mice and evaluated the impact of alpha-2-antiplasmin (α_2_AP) deficiency on the spreading and proteolytic activity of *S. aureus* skin infections. The species-selectivity of SAK was overcome by adenoviral expression of human plasminogen. Bacterial spread and density was assessed non-invasively by imaging the bioluminescence of *S. aureus* Xen36.

**Results:**

SAK-mediated plasmin activity increased the local invasiveness of *S. aureus,* leading to larger lesions with skin disruption as well as decreased bacterial clearance by the host. Even though fibrin and bacterial surfaces protected SAK-mediated plasmin activity from inhibition by α_2_AP, the deficiency of α_2_AP resulted in increased bacterial spreading. SAK-mediated plasmin also induced secondary activation of gelatinases, shown both *in vitro* and in lesions from the *in vivo* model.

**Conclusion:**

SAK contributes to the phenotype of *S. aureus* skin infections by enhancing bacterial spreading as a result of fibrinolytic and proteolytic activation.

**Electronic supplementary material:**

The online version of this article (doi:10.1186/s12866-014-0310-7) contains supplementary material, which is available to authorized users.

## Background

*Staphylococcus aureus (S. aureus)* is the leading cause of skin and soft tissue infections, both community- and hospital-acquired [1,2]. *S. aureus* is a versatile pathogen that has the intriguing capacity to modulate both the host’s coagulation and fibrinolytic system.

We and others have shown that fibrin formation induced by the bacterial prothrombin activators staphylocoagulase and von Willebrand factor-binding protein is an important virulence factor for both localized and systemic infections by *S. aureus* and is essential for abscess formation [3-6]. Most *S. aureus* strains causing infection in humans also produce staphylokinase (SAK). SAK forms an equimolar complex with human plasmin (huPli) catalyzing the further activation of plasminogen. The SAK-huPli complex is sensitive to rapid inhibition by alpha-2-antiplasmin (α_2_AP) unless it is bound to fibrin via the lysine binding sites of plasmin. This mechanism accounts for the fibrin-specificity of SAK [7,8]. Although SAK has received considerable research attention during its development as a fibrinolytic agent in cardiovascular medicine [7], few studies have investigated its relevance in *S. aureus* infection [9,10].

Streptokinase, secreted by group A, C and G streptococci, is the other well-known bacterial plasminogen activator. Like SAK, streptokinase is specific for human plasminogen (huPlg) but its action is not inhibited by α_2_AP [11]. Streptokinase is a key virulence factor and the primary determinant of the species-selectivity of group A streptococcal infections. Mortality after infection with group A streptococci is markedly increased in *huPlg* transgenic mice [12,13]. Interestingly, a subcluster of streptokinase (type 2b) has been identified that is sensitive to α_2_AP inhibition, leading to site-restricted plasminogen activation in these, mostly skin-trophic, group A streptococci [14].

The role of SAK as a potential virulence factor in *S. aureus* disease remains unresolved. SAK is present in the large majority of *S. aureus* strains causing human infection [15-24]. In contrast, *S. aureus* strains from veterinary sources commonly lack SAK production [23,24]. SAK, like streptokinase [25], thus constitutes an adaptation of *S. aureus* for human infection. The *sak* gene has a highly conserved sequence [26-29] and is carried on a bacteriophage containing other genes with an important function in immune evasion, such as complement inhibitory factors and enterotoxins [21,30]. SAK was shown to enhance the breaching of tissue barriers *in vitro* [10].

The present study aimed at evaluating if plasmin generation by SAK impacts on proteolytic activity in *S. aureus* infected skin and on the local and systemic dissemination of *S. aureus*. We further investigated if the host α_2_AP, by inhibiting the SAK-plasmin activity, reduces the virulence of *S. aureus*.

## Results

### Bacterial staphylokinase production

**Figure 1:**
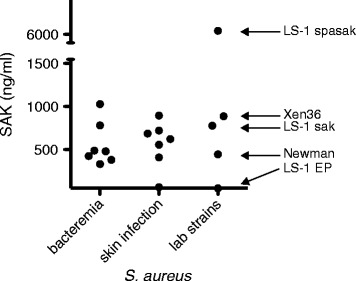
**Bacterial staphylokinase production.** Staphylokinase (SAK) production of different *S. aureus* strains after overnight culture, as assessed by ELISA. SAK secretion of the bioluminescent strain *S. aureus* Xen36 is comparable to relevant clinical *S. aureus* strains from bacteremia with cutaneous origin and from skin infection. SAK production of reference lab strains, including a SAK-negative (LS-1 *EP*) and a SAK-overproducing (LS-1 *spasak*) *S. aureus* strain are included as controls.

Supernatant of overnight cultures of the bioluminescent *S. aureus* Xen36 contained similar levels of SAK compared to lab strains and to clinical *S. aureus* strains from skin infection and from bacteremia with cutaneous origin. SAK expression by LS-1 *spasak*, which expresses SAK under the control of the *protein A* promoter, was about tenfold higher (Figure 1). Thus, *S. aureus* Xen36 is a relevant micro-organism to study the role of SAK in a skin infection model.

### Skin infection model

#### Adenoviral-mediated human plasminogen expression

**Figure 2:**
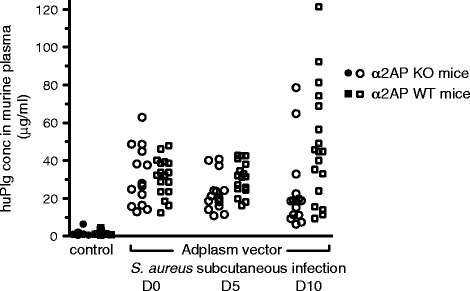
**Adenoviral-mediated human plasminogen expression.** Human plasminogen (huPlg) expression in murine plasma after administration of 5 × 10^10^ viral particles of adenoviral vector Adplasm. Day 0 is the day of subcutaneous infection with *S. aureus* Xen36, 7 to 11 days after adenoviral injection. Values from Adnull injected mice are included as negative controls. The expression of huPlg during the whole course of the subcutaneous infection experiment allows the selective interaction of staphylokinase with huPlg.

To overcome the species-selectivity of SAK for huPlg, huPlg was expressed in mice through adenoviral gene transfer. Seven to 11 days after adenoviral injection, i.e. at the start of the subcutaneous infection experiment, huPlg plasma values in Adplasm injected mice were 31.6 ± 15.5 μg/ml in α_2_AP KO and 31.5 ± 10.1 μg/ml in α_2_AP WT mice. In mice injected with the control Adnull adenoviral vector, values of huPlg were below the detection level (3 μg/ml). Human plasminogen remained present until the end of the experiment (27.3 ± 10.0 μg/ml at day 5 and 37.2 ± 29.6 μg/ml at day 10) (Figure 2). Hence, the expression of huPlg allowed for the selective interaction of SAK with huPlg throughout the course of the subcutaneous infection experiment.

#### Infectious skin lesions

**Figure 3:**
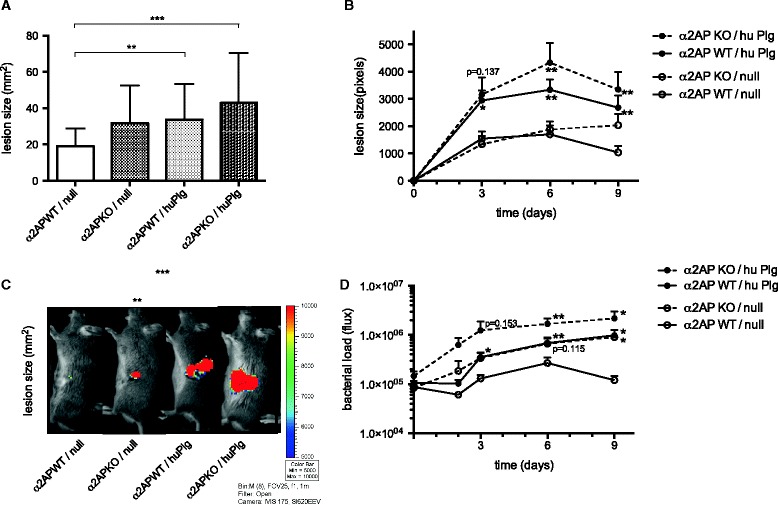
**SAK-mediated plasmin activity increased infectious skin lesion size and bacterial load. A**. Macroscopically apparent lesion size 10 days after subcutaneous inoculation with *S. aureus* Xen36. Mean and SD for skin lesions in WT/null mice (n = 12), α_2_AP KO/null mice (n = 11), WT/huPlg mice (n = 17) and α_2_AP KO/huPlg mice (n = 14), respectively. **B**. Lesion size: *S. aureus* Xen36 possesses a stable copy of the modified *Photorhabdus luminescens luxABCDE* operon. Evolution of bacterial spread was assessed non-invasively by bioluminescence image analysis of the surface area with signal > threshold. Mean and SEM for lesion size in the 4 groups. Dimensions for α_2_AP KO/huPlg mice and WT/huPlg mice are compared to WT/null mice. **C**. Examples of bioluminescence photographs of the left flank lesion for representative animals of the 4 groups at day 9. **D**. Bacterial load: signal intensity of the infectious lesion site, in photons/s through a defined region of interest, which was used for all lesions. Bacterial density analysis shows that bacterial clearance is hampered by enhanced proteolytic activity. Mean and SEM for signal intensity in the 4 groups. Bacterial loads for α_2_AP KO/huPlg, α_2_AP KO/null and WT/huPlg mice are compared to WT/null mice. *denotes P < 0.05, **P < 0.01, ***P < 0.001.

After subcutaneous inoculation with *S. aureus* Xen36, macroscopic lesion size was significantly larger in huPlg expressing mice compared to control wild type mice (33.6 ± 19.6 mm^2^ in WT/huPlg mice (n = 17) vs. 19.2 ± 9.7 mm^2^ in WT/null mice (n = 12) at day 10, P < 0.01) (Figure 3A). The constitutive luciferase expression of *S. aureus* Xen36 also allowed for non-invasive monitoring of the spreading and density of the bacteria over time (illustrated in Figure 3C). Expression of huPlg increased both bacterial spreading (P < 0.05 on day 3, P < 0.01 on day 6 and 9) and bacterial load (P < 0.05 on day 3 and 9, P < 0.01 on day 6) early in the course of infection (Figures 3B and 3D).

Compared to wild type mice, bacterial spreading in α_2_AP KO mice was similar in the early stages of the infection, but was more pronounced at day 9 (P < 0.05) (Figure 3B), resulting in an increase in macroscopic lesion size at day 10 (31.8 ± 20.9 mm^2^ in α_2_AP KO/null mice (n = 11) vs. 19.2 ± 9.7 mm^2^ in WT/null mice (n = 12), P = 0.078) (Figure 3A). Bacterial density was higher in α_2_AP KO mice compared to wild type mice (P = 0.115 on day 6, P < 0.05 on day 9) (Figure 3D). In an additional experiment, α_2_AP KO or WT mice were infected with *S. aureus* LS-1 *EP*. We observed similar initial fibrin deposition in the abscess periphery in both groups at day 1 (Additional file 1), consistent with a normal capacity for fibrin formation in α_2_AP KO plasma ex vivo (data not shown). However, at a later time point, less fibrin was observed in α_2_AP KO mice, as shown in Additional file 1.

The largest lesion size was observed in the α_2_AP KO mice with human plasminogen expression (42.9 ± 27.4 mm^2^ in α_2_AP KO/huPlg (n = 14) vs. 19.2 ± 9.7 mm^2^ in WT/null (n = 12), P < 0.001) (Figure 3A). The spreading of the bacteria was more pronounced from early in the course of infection (P = 0.137 on day 3, P < 0.01 on day 6 and 9) (Figure 3B). Also the intensity of the bioluminescence, which relates to the bacterial density, was the highest in the α_2_AP KO/huPlg group (P = 0.153 on day 3, P < 0.01 on day 6, P < 0.05 on day 9) (Figure 3D).

The assessment of systemic spread of *S. aureus*, by quantifying bacterial load in spleen and kidney, did not differ significantly between the 4 groups. At day 10, there were 2 mice in the α_2_AP KO/huPlg group with distant infection in spleen and/or kidney, 2 mice in the α_2_AP KO/null group, 1 mouse in the WT/huPlg group and none in the WT/null group.

In the first series of experiments, we observed that the differentiation between the groups both in macroscopic phenotype (open/closed lesion) and in bioluminescence lesion size and intensity became apparent early in the course of infection. Therefore, to assess macroscopic and microscopic phenotype and proteolytic activity, mice were sacrificed at day 3 in a subsequent set of experiments.

**Figure 4:**
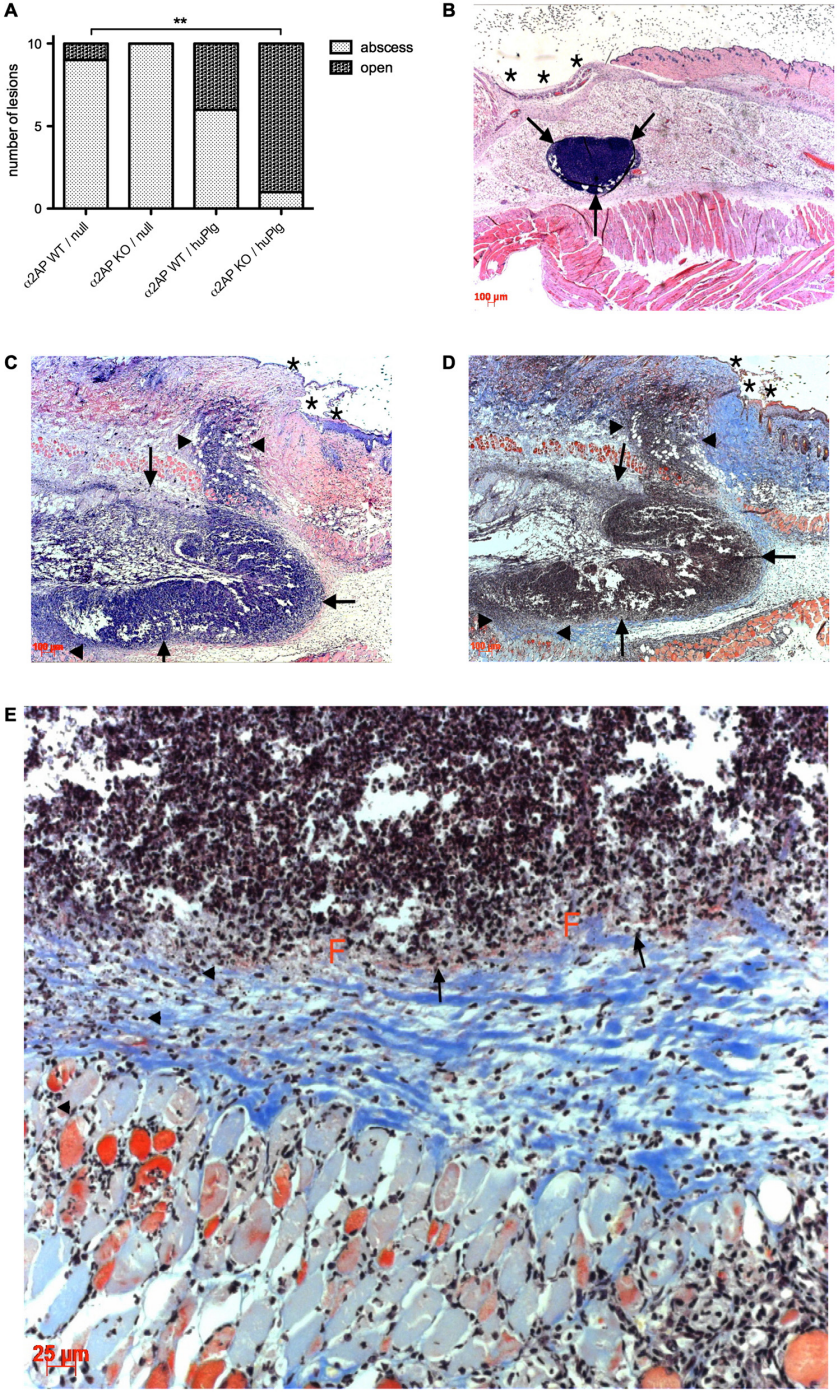
**Infectious skin lesions at day 3 after subcutaneous infection with** ***S. aureus*** **Xen36. A**. Macroscopic aspect of day 3 lesions. More open lesions with skin rupture are observed in α_2_AP KO/huPlg mice, compared to WT/null mice (P <0.01). **B**. Hematoxyllin-eosin staining of lesional skin from WT/null mouse showing a small abscess collection (arrows) without disruption of overlying skin (*). **C**. Hematoxyllin-eosin staining of lesional skin from α_2_AP KO/huPlg mouse showing a large, less well-defined abscess collection (arrows), with extension (arrowheads) from the initial infection site both towards the overlying skin (*) (with resulting skin disruption and crust formation) and towards the underlying subdermal tissue and muscularis. **D-E**. Martius Scarlet Blue staining of the same lesion reveals a zone of fibrin deposition (F, red) at the periphery of the initial abscess site (arrows), but the infection has spread past this border of fibrin, through collagen fibers (blue), into underlying tissue layers (arrowheads).

Macroscopical assessment confirmed closed abscesses in WT/null mice, compared to more diffusely spread lesions with skin rupture in huPlg expressing mice (P < 0.01) (Figure 4A-C).

Histopathologic analysis of lesional skin sections from α_2_AP KO/huPlg mice showed, apart from breaching of skin, penetration of infection starting from the initial infection site, past a peripheral fibrin zone, into subdermal tissue layers (Figure 4C-E).

### Mechanism of SAK action in *S. aureus* skin infections

#### SAK is species-selective and fibrin-specific

**Figure 5:**
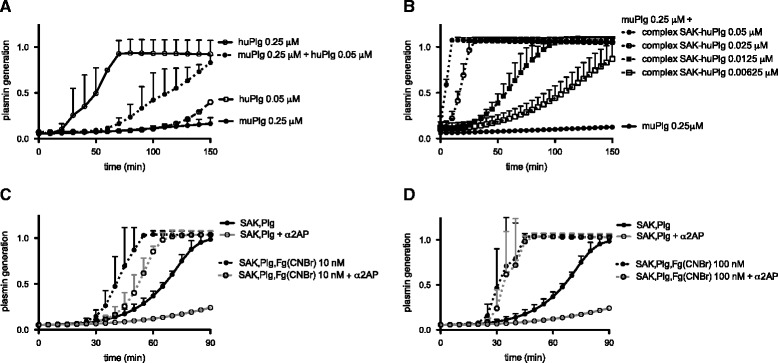
**Species-selectivity and fibrin-specificity of staphylokinase. A**. Species-selectivity of staphylokinase (SAK) for human plasminogen (huPlg). Plasmin generation by adding SAK (6.25 nM) to either huPlg (0.25 μM), murine plasminogen (muPlg 0.25 μM), or a mixture of muPlg (0.25 μM) with huPlg (0.05 μM or 20% of the muPlg concentration, comparable to the level of huPlg in murine plasma after adenoviral-mediated huPlg expression). Plasmin generation was quantitated by conversion of the chromogenic substrate S-2403 and assessed in a microtiter plate ELISA reader at 405 nm. Mean and SD from 3 independent experiments. **B**. Plasmin generation by a preformed equimolar mixture of SAK with huPlg, added to muPlg (0.25 μM), highlighting that low levels of huPlg in a background of muPlg can induce efficient SAK-dependent plasmin generation. Mean and SD from 3 independent experiments. **C-D**. The complex of SAK with human plasmin (SAK-huPli) is protected from inhibition by alpha-2-antiplasmin (α_2_AP) in the presence of fibrin analogues. Plasmin generation by SAK (6.25 nM) in a mixture of muPlg (0.25 μM) and huPlg (0.05 μM); with or without α_2_AP (0.125 μM) and either in the absence or presence of Fg(CNBr) (**C**. and **D**. for Fg(CNBr) 10 nM and 100 nM, respectively). Mean and SD from 3 independent experiments.

The observations in the subcutaneous infection model can be explained by the SAK-mediated plasmin generation. To this end however, the values of huPlg achieved after adenoviral huPlg expression in mice, should be able to rescue the species-selectivity of SAK in a murine model. SAK induced rapid plasmin generation if added to huPlg but not with murine plasminogen (muPlg) (Figure 5A). However, activation of muPlg was observed in the presence of SAK and trace amounts of human plasminogen (Figure 5A). Also, addition of a preformed SAK-huPli complex triggered secondary activation of muPlg, as illustrated in Figure 5B. For all further experiments, a mixture of muPlg (0.25 μM) and huPlg (0.05 μM) was used to reflect the *in vivo* conditions of partial huPlg expression against a background of muPlg.

SAK-mediated plasmin generation was further enhanced in the presence of fibrin, as CnBr-digested fibrinogen fragments (Fg(CNBr)) (Figure 5C-D) or as solid fibrin (data not shown). For convenience, Fg(CNBr) was used in further experiments, as an accepted soluble alternative to solid fibrin. This increase in plasmin generation in the presence of fibrin can be explained by a reduced inactivation of fibrin-associated SAK-huPli complex by α_2_AP. As shown in Figure 5C, α_2_AP inhibited SAK-induced plasmin generation in control conditions, but had little effect on SAK-induced plasmin generation in the presence of Fg(CNBr) (A_405,60min_ of 0.853 ± 0.058 vs. 0.121 ± 0.005 for SAK + Plg + α_2_AP, P < 0.01). Higher concentrations of Fg(CNBr) completely protected SAK-induced plasmin from inhibition by α_2_AP (A_405, 60min_ of 1.036 ± 0.039 vs. 0.121 ± 0.005 for SAK + Plg + α_2_AP, P < 0.001) (Figure 5D).

#### Bacterial cell surface protects SAK from α_2_AP inhibition

**Figure 6:**
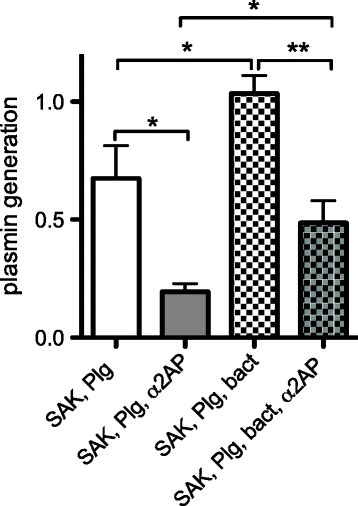
**SAK-huPli complex is protected from inhibition by α**_**2**_ **AP in the presence of** ***S. aureus*** **bacterial surfaces.** *S. aureus* bacterial surfaces enhance SAK-mediated plasmin generation and partially protect the SAK-huPli complex from inhibition by α_2_AP. Plasmin generation by SAK (6.25 nM) in a mixture of murine (0.25 μM) and human (0.05 μM) plasminogen, with or without α_2_AP (0.125 μM) and in the absence or presence of washed SAK-negative *S. aureus* LS-1 *EP* (OD_600_ 2.0, 15% vol/vol). Plasmin generation was quantitated by conversion of the chromogenic substrate S-2403 and assessed in a microtiter plate ELISA reader at 405 nm. Mean and SD from 3 independent experiments. *denotes P < 0.05, **P < 0.01.

We also studied if, besides fibrin, bacterial surfaces could protect the SAK-huPli complex from α_2_AP inhibition. Indeed, the presence of bacteria increased plasmin generation following addition of SAK (A_405, 90min_ 1.035 ± 0.076 vs. 0.675 ± 0.138, P < 0.05). Addition of murine α_2_AP significantly reduced plasmin generation by ≈ 70% to A_405, 90min_ of 0.195 ± 0.034 (P < 0.05), whereas in the presence of bacteria, α_2_AP only led to a ≈ 50% reduction in plasmin generation (A_405, 90 min_ = 0.487 ± 0.094, P < 0.01) (Figure 6). We used a SAK-negative *S. aureus* strain (LS-1 *EP*) for this subset of experiments to eliminate confounding by SAK production during the course of the experiment. However, similar results were observed for SAK-positive *S. aureus* Xen36, which was the strain used in animal experiments. Comparable results were also obtained when heat-killed instead of viable *S. aureus* was used (data not shown).

#### SAK-huPli complex activates gelatinases

Plasmin has a broad proteolytic spectrum that includes extracellular matrix proteins. However, plasmin is also known to activate gelatinases, which can cause secondary proteolytic activity. In order to assess whether gelatinase activation contributes to the observed bacterial spreading, we measured the activation of gelatinases by SAK-huPli in murine skin extracts.

**Figure 7:**
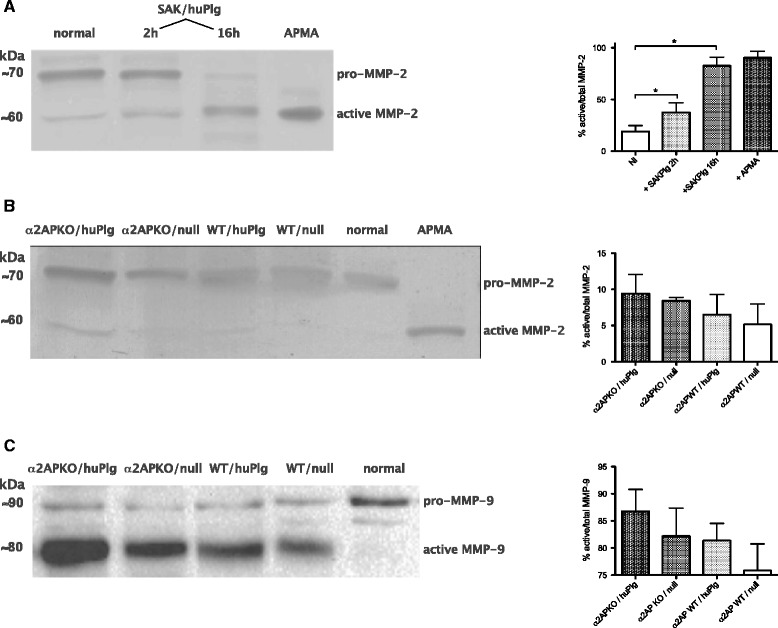
**Gelatinase activation by SAK in skin tissue. A**. Gelatin zymogram showing pro-MMP-2 and active MMP-2 in murine skin and subcutaneous tissue protein extract after incubation with staphylokinase (SAK) and human plasminogen (huPlg). Activation with APMA, a chemical MMP-activator, was used as a positive control. Quantitation of MMP-2 activation (active/total), data are mean ± SD from 3 independent experiments. *denotes P < 0.05. **B**. Gelatin zymogram showing pro-MMP-2 and active MMP-2 in day 3 lesional skin samples. A sample from normal skin and an APMA-activated normal skin extract are included as controls. Quantitation of MMP-2 activation (active/total), data are mean ± SD from 3 independent experiments. P for trend <0.05. **C**. MMP-9 activation in day 3 lesional skin samples, assessed by Western blot. A sample from normal skin is included as control. Quantitation of MMP-9 activation (active/total), data are mean ± SD from 3 independent experiments. P for trend <0.05.

Addition of SAK and huPlg to extracts of murine skin led to activation of pro-MMP-2 (Figure 7A). In line with the low expression of MMP-9 in normal non-inflamed skin, pro-MMP-9 and active MMP-9 could not be clearly identified on these zymograms of murine skin. However, we did observe MMP-9 activation in HT1080 cell culture supernatant after incubation with SAK and huPlg (data not shown).

Zymograms of day 3 lesions (2 representative lesions per group) showed a higher ratio of active/total MMP-2 in α_2_AP KO/huPlg mice, compared to control WT/null mice (Figure 7B). Increased MMP-9 expression and activity was observed in all infected skins compared to normal skin, with a similar higher ratio of active/total MMP-9 in the α_2_AP KO/huPlg group compared to control WT/null mice (Western blot, Figure 7C).

## Discussion

We evaluated the role of SAK-mediated plasminogen activation in a subcutaneous *S. aureus* skin infection model in mice. Our results demonstrate that plasmin generation by SAK increased spreading and hampered bacterial clearance of *S. aureus* in infected skin, increased tissue proteolytic MMP activity, and resulted in more pronounced tissue damage, as noted by more open skin lesions. These findings were enhanced in the absence of the plasmin inhibitor α_2_AP, demonstrating a role for host α_2_AP in the containment of *S. aureus* infections.

Strengths of our model are the use of a *S. aureus* strain derived from a human infection in healthy mice, and the non-invasive follow-up of bacterial spreading by bioluminescence. The use of an adenoviral vector encoding huPlg resulted in plasma huPlg levels similar to those attained in transgenic *huPlg* mice [12]. Our *in vitro* data confirm that these huPlg concentrations in mice are sufficient to overcome the species-selectivity of SAK and to mimic the phenotype of SAK-mediated huPlg activation in the subcutaneous infection model.

Our results are in line with previous results showing that spreading through tissue barriers can be mediated by SAK activity [10]. Plasmin activity generated by SAK can degrade several extracellular matrix components, but not collagens [31]. Yet, we show that SAK-mediated plasmin activity can lead to secondary activation of gelatinases in mouse skin extracts, which may contribute to the proteolytic activity necessary for dissemination through tissue.

Although we observed more proteolytic activity and increased lesion dimensions in α_2_AP KO/huPlg groups, we did not observe systemic spread nor mortality after subcutaneous inoculation of *S. aureus*. However, systemic dissemination was common when using a neutropenic mouse model of *S. aureus* skin infection, as recently shown [10]. This is in agreement with the clinical observation that skin infections by *S. aureus* infrequently lead to systemic dissemination, unless there is an underlying vulnerability of the patient. In immunocompetent mice (and patients), staphylococcal skin and soft tissue infections are mainly characterized by abscess formation, and bacteria that do reach the systemic circulation or distant organs are rapidly cleared.

The predominantly local effect of SAK-mediated plasmin activity without affecting the severity of systemic symptoms is also in contrast with the findings by Sun *et al.* [12], demonstrating that streptokinase increased mortality of Group A streptococcal infection in a skin infection model in *huPlg* transgene mice. Importantly, streptokinase is insensitive to α_2_AP inhibition, in contrast with the SAK-huPli complex that is rapidly inactivated by α_2_AP. Our results confirm previous findings that fibrin protects the SAK-huPli complex from inactivation; and a similar protective effect was noted from bacterial surfaces [8,32,33]. This may explain why SAK-mediated plasmin has more localized effects compared to streptokinase-induced plasmin. SAK-induced proteolysis is thus confined to the immediate surroundings of the site of infection, where high concentrations of fibrin and bacteria prevent inactivation, but is rapidly neutralized further away from the abscess site. In contrast, streptokinase-activated plasmin may have a larger potential for systemic effect because of its resistance to α_2_AP.

To explore whether inhibition by α_2_AP accounts for the more localized effect of SAK compared to streptokinase, we studied the impact of the genetic absence of α_2_AP on the characteristics of *S. aureus* skin infection. Indeed, α_2_AP KO was associated with larger lesion sizes, compatible with a protective role of α_2_AP in tempering the proteolytic effect of SAK-mediated plasmin. The larger lesion size in α_2_AP KO/null mice, lacking huPlg expression, is likely explained by activation of murine plasminogen, bound to surface plasminogen receptors of *S. aureus*, by host plasminogen activators. Different surface plasminogen receptors have been described in *S. aureus*, such as α-enolase, inosine 5’-monophosphate dehydrogenase, ribonucleotide reductase subunit 2, triose phosphate isomerase, surface immunoglobulin-binding protein and extracellular fibrinogen-binding protein [33-35]. These surface plasminogen receptors are not selective for huPlg and constitute a common theme across different bacterial and fungal organisms for the degradation of extracellular matrix and immune evasion [35,36]. The resulting surface-bound plasmin activity is less sensitive to inhibition by plasma protease inhibitors, hence a slower but consistently larger expansion of the lesion occurs in the α_2_AP KO/null group compared to WT/null controls*.* The role of host α_2_AP in limiting bacterial spreading was confirmed in additional experiments where a SAK-negative strain (*S. aureus* LS-1 *EP*) was used to infect α_2_AP KO and α_2_AP WT mice. As α_2_AP gene deficiency does not impair fibrin generation, the initial fibrin deposition surrounding the abscess was comparable in both groups. However, at a later time point the absence of the main plasmin inhibitor led to increased dissolution of the peripheral fibrin sheath. Although there was a trend towards higher numbers of systemic infection in α_2_AP KO mice (4/25 vs 1/29 WT mice), we could not demonstrate a strong effect of α_2_AP KO on systemic spreading. Although α_2_AP is the predominant plasmin inhibitor, other plasma inhibitors of fibrinolysis such as alpha-2-macroglobulin may explain the absence of systemic spread in α_2_AP KO/huPlg mice [32].

Interestingly, we also observed increased bacterial loads in the presence of huPlg and/or in the absence of α_2_AP, demonstrating that bacterial induced plasmin generation helps to evade bacterial clearance by the host. It has been shown previously that SAK binds to and inactivates human defensins, part of the innate immune defense against bacteria [37]. SAK-induced plasmin activity can degrade opsonizing complement components IgG and C3b [38], thus protecting *S. aureus* from phagocytosis.

The production of SAK, a highly fibrin-specific plasminogen activator [7,8], is particularly intriguing as *S. aureus* also triggers fibrin formation through coagulase activity. The role of staphylocoagulase-mediated fibrin deposition in abscess formation is well established [3]. *S. aureus* also possesses different binding proteins which interact with fibrin(ogen) and extracellular matrix proteins [39]. It remains to be resolved how coagulase-mediated fibrin deposition and SAK-mediated fibrinolysis cooperate to promote *S. aureus* virulence. Coagulase activity and the resulting fibrin have been shown to shield *S. aureus* from leukocytes, promoting early replication and persistence. In this view, SAK allows *S. aureus* to generate fibrinolytic activity that is protected by both the staphylothrombin- and thrombin-generated fibrin from rapid neutralization by host protease inhibitors. The resulting plasmin activity can degrade the host’s fibrin as well as the *S. aureus*-mediated fibrin and allow subsequent spreading of the growing bacterial colony. How *S. aureus* differentially regulates coagulase and SAK activity remains unknown. Interestingly, SAK expression is under control of the *agr* quorum sensing system, suggesting that proteolytic activity is modulated by bacterial density [40]. A regulated expression of SAK may also explain the observed decreased virulence of genetically engineered *S. aureus* strains with SAK overproduction, not under control of its own promotor, as continuous and high-level SAK production will interfere with coagulase activity as a central virulence factor of *S. aureus* [9,10].

## Conclusions

We show that SAK-mediated proteolytic activity in *S. aureus* infected skin facilitates local spreading, increases tissue damage of skin and reduces bacterial clearance by the host. The underlying mechanisms involve protection of the SAK-huPli complex associated with fibrin or bacterial surfaces, from rapid inhibition by α_2_AP. In turn, active SAK-huPli may activate gelatinases, further promoting degradation of the extracellular matrix. Overall, this study shows the role of subversion of the host fibrinolytic system by SAK-producing *S. aureus* in migration through tissue barriers.

## Methods

### Bacterial strains

All animal experiments were performed with *S. aureus* Xen36 (Caliper Life Sciences, Hopkinton, USA), a bioluminescent strain derived from the parental strain *S. aureus* ATCC 49525 (Wright), a clinical isolate from a patient with bacteremia. *S. aureus* Xen36 possesses a stable copy of the modified *Photorhabdus luminescens luxABCDE* operon at a single integration site on a native plasmid.

Clinical strains were collected from patients at the University Hospitals Leuven and originated from either skin infection or bacteremia secondary to a skin infection with *S. aureus*. Laboratory strains included *S. aureus* Newman and 3 different congenic *S. aureus* LS-1 variants with different SAK expression (LS-1 *EP*, LS-1 *sak* and LS-1 *spasak*) [9].

All strains were stored in Brain Heart Infusion (BHI) with glycerol at −80°C. Before use, strains were grown overnight in Tryptic Soy Broth (TSB) at 37°C in aerobic conditions. For subcutaneous infection, overnight cultures were washed twice with PBS and diluted in PBS to an optical density at 600 nm (OD_600_) of 2.0, corresponding to 2 × 10^9^ CFU (colony forming units)/mL. CFU counts were confirmed by quantitative plating of the inoculum for each experiment. SAK production was confirmed in the supernatant of overnight cultures using an in-house developed sandwich ELISA (MA-S20D11 + MA-S25F6/PA-RaSTAN ELISA).

### Animal experiments

All animal experimental procedures were approved by the Ethics Committee of the KU Leuven.

#### Mouse strains

α_2_AP knock-out mice in C57BL/6 - S129Vj background and their littermate wild type controls were used [41].

#### Adenoviral expression of huPlg

Because of the selectivity of SAK for huPlg, we studied the role of SAK in a subcutaneous *S. aureus* infection model after huPlg overexpression through adenoviral gene transfer. An E1E3E4-deleted adenoviral vector inducing hepatocyte-specific expression of huPlg (Adplasm) was used. In this vector, the expression of huPlg is under control of the alpha-1-antitrypsin promoter and four copies of the human Apo E enhancer [42]. Adnull, a similar adenoviral vector lacking an expression cassette was used as control [43].

Male mice of 5-9 weeks were injected via the tail vein with 5 × 10^10^ particles of either Adplasm or Adnull vector, 7-11 days prior to the subcutaneous infection. Hence, 4 different groups of mice were studied, which are identified as α_2_AP KO/huPlg, α_2_AP KO/null, WT/huPlg and WT/null, respectively.

#### Quantification of huPlg

Plasma concentrations of huPlg were quantitated by ELISA and by a functional test specific for huPlg, allowing measurement of huPlg concentrations in murine plasma. The in-house developed sandwich ELISA, based on the antibodies MA-42B12B4B2D and MA-34D3D10-HRP does not cross-react with murine plasminogen. The functional assay is based on the species-selectivity of streptokinase, and measures plasmin generation with a chromogenic substrate (S-2403, Chromogenix, Milano, Italy) after addition of an excess of streptokinase (1000 IU/mL, Streptase, CSL Behring, Marburg, Germany).

Preliminary experiments showed reliable and stable expression of huPlg from day 7 up to 1 month (day 43) after injection of adenoviral vector.

#### Skin infection model

Approximately 1 week post adenoviral injection, mice were anesthesized with isoflurane and injected subcutaneously in each flank with 100 μL containing 2 × 10^8^ CFU of *S. aureus* Xen36. Blood samples were collected on citrate (3.2% sodiumcitrate, 10% vol/vol) by retro-orbital puncture, at day 0 (before infection) and day 5. At day 10, animals were killed by heart puncture under high dose ketamine/xylazine anesthesia. Largest diameter *(a)* and orthogonal diameter *(b)* of skin lesions were measured with a caliper, and skin lesion areas were calculated ((π/4)*ab*). Lesions were then excised for histological analysis. Dimensions of lesions from left and right flank were averaged per individual mouse. Spleen and kidneys were also collected for analysis of bacterial load.

One mouse died on day 4 in the α_2_AP KO/null group, this subject was not included in analysis.

In a supplementary experiment, to study the effect of host α_2_AP in staphylococcal skin infection, this subcutaneous infection model was carried out with SAK-negative *S. aureus* LS-1 *EP* in α_2_AP KO or WT mice without previous adenoviral injection.

#### Bioluminescence imaging

Non-invasive follow-up of the local spreading of *S. aureus* Xen36 was performed by means of bioluminescence imaging of the luciferase signal with a cooled CCD camera (IVIS 100, Xenogen, Perkin-Elmer Company, Alameda, USA). Mice were sedated with isoflurane and imaging was performed for each lesion with an exposure time of 60 s. Signal intensity was calculated with Living Image 2.5 analysis software (Xenogen) and denotes photons per second through a defined region of interest (ROI), corresponding to the infectious lesion. The same ROI was used for all infectious lesions in all animals. Preliminary experiments showed a correlation between bioluminescence signal intensity and bacterial load of *S. aureus* Xen36 (Pearson r = 0.965, P = 0.0001). A fixed threshold was chosen for all images and quantitation of the lesion size (pixels with signal above threshold) was performed with Image J software (Image J, NIH, Bethesda, USA).

#### Histology

Paraffin-embedded tissue samples were used to prepare 10 μm thick sections. Routine histopathologic stainings with hematoxyllin-eosin or Martius Scarlet Blue (for fibrin) were performed.

### Study of plasminogen activation by SAK

We studied the activation of plasminogen (human, murine, or a mixture of both) by SAK in the absence or presence of α_2_AP, fibrin and *S. aureus* bacteria. Human and murine plasminogen were isolated from plasma by lysine Sepharose affinity chromatography, as described previously [44]. SAK variant TS-162 was previously described [45]. Murine α_2_AP was obtained from Abcam (Cambridge, UK). SAK, α_2_AP and plasminogen were diluted in 0.1M sodium phosphate buffer, pH 7.4, containing 0.05 M NaCl and 0.01% Tween. Solid fibrin clots were formed upon addition of bovine thrombin (1 U/mL) to human fibrinogen (200 μg/mL in 0.05 M Tris-HC1 buffer, pH 7.4, containing 0.038 M NaCl and 0.01% Tween 80) (Calbiochem, EMD Millipore, Billerica, USA) (30 min, 37°C). CNBr-digested murine fibrinogen (Fg(CNBr)) was prepared as published [46]. In some experiments, *S. aureus* bacteria (OD_600_ 2.0, 15% vol/vol; live or heat-killed at 60°C for 1h) were used in the reaction mixture. In this case, bacteria were pelleted by centrifugation before read-out of the absorbance at 405 nm (A_405_). Hydrolysis of the chromogenic substrate S-2403 was used to monitor plasmin activity in a Bio-TEK microtiter plate reader (Bio-TEK, Winooski, USA).

### Blotting techniques

Gelatin zymography was used to study activation of the gelatinase subfamily of matrix metalloproteinases (MMPs) by the SAK-human plasmin complex (SAK-huPli). To this end, tissue extracts of skin and subcutaneous tissue from healthy C57BL/6 mice were prepared as described [47,48]. Briefly, tissue samples were snap frozen in liquid nitrogen. Protein extraction was performed by homogenization with glass beads in FastPrep24 (MP Biomedicals, Santa Ana, USA) in the presence of extraction buffer (10 mM sodium phosphate, pH 7.2, containing 150 mM NaCl, 1% Triton X-100, 0.1% SDS, 0.5% sodium deoxycholate, and 0.2% NaN_3_). After centrifugation, the protein concentration of the supernatant was determined with the Bradford assay (Bio-rad, Hercules, USA). The skin extracts were incubated (2 h or 16 h, 37°C) with a mixture of 1.1 μM huPlg and SAK (in a 1:10 molar ratio to huPlg).

Zymographic analysis of gelatinase activity was performed on 10% Tris-glycine gels containing 0.1% gelatin (Novex, Life Technologies, Carlsbad, USA).

We used MMP-containing medium from a HT1080 fibrosarcoma cell line (Sigma-Aldrich, St. Louis, USA) and activation by APMA (4-amino-phenyl-mercuric acetate, a chemical MMP-activator, Sigma-Aldrich) as a reference.

Preliminary experiments showed that plasmin also generated a gelatinolytic band on zymography. To irreversibly inactivate plasmin prior to loading, samples were treated with a 100-fold molar excess of D-Val-Phe-Lys-chloromethylketone, dihydrochloride (Sigma-Aldrich) (15 min, room temperature).

The lysis of the substrate gel (area × intensity) was quantitated by image analysis (Image J) [47].

Western blotting for murine MMP-2 and MMP-9 was performed using a rabbit polyclonal antibody (NB200-193 for MMP-2, Novus Biologicals, Cambridge, UK and ab38898 for MMP-9, Abcam).

### Statistical analysis

All calculations were performed using GraphPad Prism 5.0b (GraphPad Software, San Diego, USA). Data were tested for normality and appropriate tests were used to compare continuous variables between groups (t-test if normal distribution, Mann-Whitney U test if not). For bioluminescence data, values were compared at definite time points with 1-way ANOVA, using t-test or Mann-Whitney U test as post-test. For comparison of bioluminescence data over different time points and between groups, 2-way ANOVA was used. For plasmin generation experiments, A_405_ values were compared by repeated measures 1-way ANOVA, using paired t-tests as post-test. Quantitative data from blotting experiments were analyzed for trend using linear regression. P-values < 0.05 were considered statistically significant. In graphs, *denotes P < 0.05, **P <0.01, ***P < 0.001.

## Additional file

Additional file 1:
**Host α**_**2**_ **-antiplasmin (α**_**2**_ **AP) protects against extension of** ***S. aureus*** **skin infection.** Martius Scarlet Blue staining of representative lesions from α_2_AP KO and α_2_AP WT mice, infected with *S. aureus* LS-1 *EP*. In both genotypes, a peripheral zone of fibrin (F, red), surrounding the abscess, can be appreciated on day 1. On day 3 however, less fibrin is observed in the abscess periphery of α_2_AP KO mice, likely reflecting the disbalance between (normal) fibrin deposition and (uninhibited) fibrinolysis at this later time point. Hence, α_2_AP gene deficiency impairs sustained local containment of *S. aureus* infection.

## Abbreviations

*S. aureus*: *Staphylococcus aureus*
SAK: staphylokinase
α_2_AP: alpha-2-antiplasmin
huPlg: human plasminogen
huPli: human plasmin
muPlg: murine plasminogen
MMP: matrix metalloproteinase
