# Supplementary figures and images for: Plasminogen activation by staphylokinase enhances local spreading of S. aureus in skin infections
Source: BMC Microbiol. 2014 Dec 17;14:310. doi: 10.1186/s12866-014-0310-7 (PMC4274676; doi:10.1186/s12866-014-0310-7)

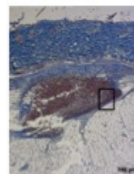

$\alpha_2$ AP WT

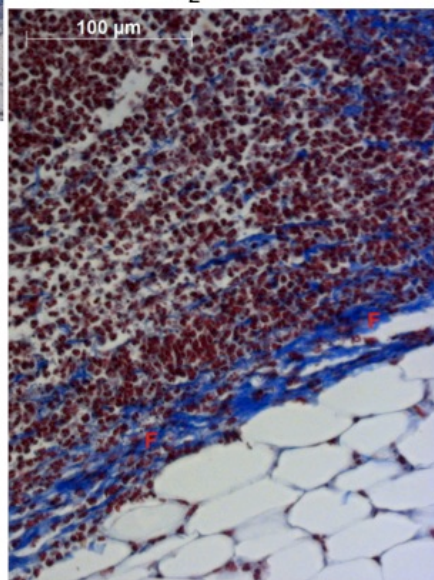

$\alpha_2$ AP KO

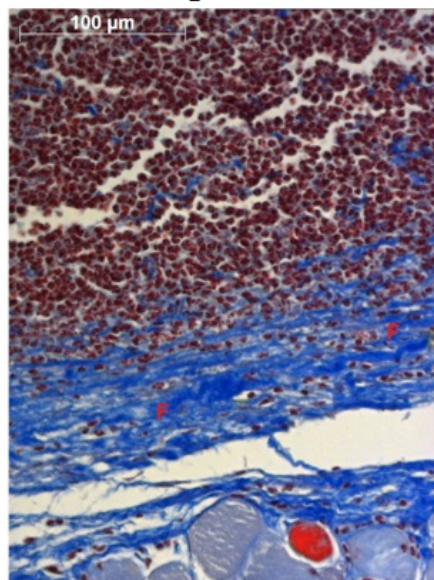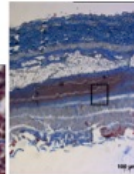

D  
A  
Y  
1

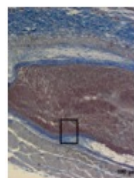

D  
A  
Y  
3

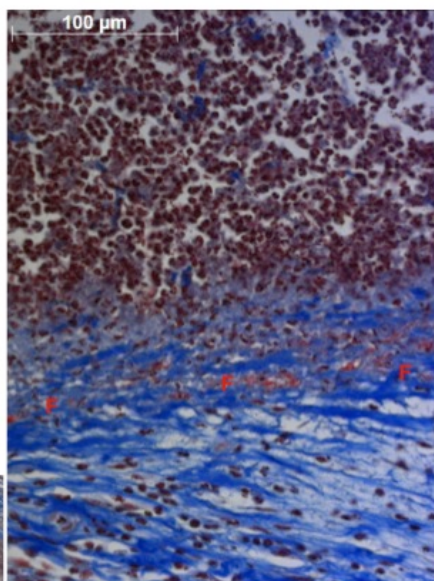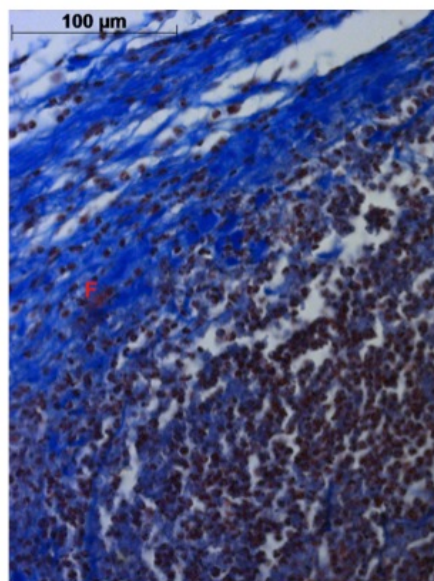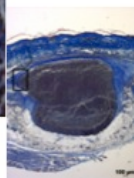

Supplement: Additional file 1: — Host α 2 -antiplasmin (α 2 AP) protects against extension of S. aureus skin infection. Martius Scarlet Blue staining of representative lesions from α2AP KO and α2AP WT mice, infected with S. aureus LS-1 EP. In both genotypes, a peripheral zone of fibrin (F, red), surrounding the abscess, can be appreciated on day 1. On day 3 however, less fibrin is observed in the abscess periphery of α2AP KO mice, likely reflecting the disbalance between (normal) fibrin deposition and (uninhibited) fibrinolysis at this later time point. Hence, α2AP gene deficiency impairs sustained local containment of S. aureus infection. [file 12866_2014_310_MOESM1_ESM.pdf]
